# The dissociation between pathological caloric testing and a normal video head impulse test helps differentiate between Menière’s disease, vestibular migraine, and other vestibular disorders: a confirmatory study in a large cohort of 2,101 patients

**DOI:** 10.3389/fneur.2024.1449261

**Published:** 2024-08-14

**Authors:** Vergil Mavrodiev, Michael Strupp, Anne-Sophie Vinck, Raymond van de Berg, Louisa Lehner

**Affiliations:** ^1^Department of Neurology, LMU University Hospital, Munich, Germany; ^2^German Center for Vertigo and Balance Disorders, LMU University Hospital, LMU Munich, Munich, Germany; ^3^Department of ENT, AZ Sint-Jan Brugge AV, Brugge, Belgium; ^4^Department of Otorhinolaryngology and Head and Neck Surgery, Division of Vestibular Disorders, Maastricht University Medical Center, Maastricht, Netherlands

**Keywords:** vertigo, Menière’s disease, vestibular migraine, video head impulse test, caloric testing, retrospective analysis, dissociation

## Abstract

Vestibular migraine (VM) and Menière’s disease (MD) are characterized by episodes of vertigo of similar duration. It is well known that differentiation between both diseases is not always possible based only on the patient history, physical examination, and audiological testing. In addition, the quantification of the vestibular function can also be helpful since, among patients with MD, there is often a dissociation between a normal/pseudo-normal video head impulse test (vHIT) and reduced caloric testing. The goal of this confirmatory study was to determine the sensitivity, specificity, and positive and negative predictive values (PPV and NPV) of this dissociation to differentiate between MD and VM as well as between MD and other vestibular diseases. We performed a retrospective analysis of 2,101 patients. The examination group consisted of 1,100 patients; of these, 627 (57%) had MD according to the diagnostic criteria of the Bárány Society and 473 (43%) had VM. The comparison group consisted of 1,001 patients with other peripheral, central, or functional vestibular disorders. Statistical analysis revealed the following findings for the dissociation: MD vs. VM: specificity: 83.5%, sensitivity: 58.9%, PPV: 82.6%, and NPV: 60.5%, and MD vs. all other vestibular disorders (VM plus others): specificity: 83.5%, sensitivity: 58.9%, PPV: 60.3%, and NPV: 82.7%. The dissociation between a normal vHIT and a reduced caloric response is due to the high specificity and PPV suited for the differentiation between MD and VM. This part of the study confirms previous findings in a large cohort of patients. When it comes to differentiating between MD and all observed vestibular disorders, if there is no dissociation, the diagnosis of MD is unlikely.

## 1 Introduction

Differentiating episodic vestibular disorders can be a challenge for any clinician, but it is crucial to ensure specific treatment. In particular, the differentiation between Menière’s disease (MD) (1) and vestibular migraine (VM) (2) is important because they share many similarities in terms of the duration of the symptoms and accompanying signs and symptoms. In typical presentations, the presence of headache, other migraineous symptoms, and history of migraine vs. hearing impairment differentiates well between the two diseases (3). On the other hand, there are also atypical forms of presentation. Especially in the early stages, approximately one-third of MD patients do not experience any auditory symptoms (4). Similarly, VM patients do not experience headaches in approximately 30% of all episodes (5, 6) and can also show an impairment of hearing (7). Finally, there are also overlap syndromes, i.e., patients fulfill the diagnostic criteria for both diseases (8).

This diagnostic clinical dilemma parallels that we do not know the exact pathophysiology and etiology of either VM (9) or MD (10, 11). It is also assumed that there is a link between both diseases (12, 13). This is reflected in many findings, for instance, the demonstration of endolymphatic hydrops in patients with VM (14), the assumption of a parallel activation of vestibular and meningeal nociceptive pathways (9, 13), and the probable role of calcitonin gene-related peptide (CGRP) in both diseases (15, 16).

Several studies demonstrated a normal vHIT and reduced caloric response (17–23) in patients with MD. This “dissociation” might serve as a diagnostic marker for MD (19). One hypothesis to explain the dissociation is that the reduced caloric excitation in MD is a result of an enlargement of the membranous duct in the hydropic labyrinths (22). This concept has been supported by animal models with similar findings to those seen in MD patients (24).

This study aimed to investigate the diagnostic significance of a normal vHIT and pathological caloric testing to (a) differentiate patients with MD from those with VM with a confirmatory approach and (b) differentiate patients with MD from patients with other vestibular disorders in a large cohort of 2,101 patients.

## 2 Methods

**Figure 1:**
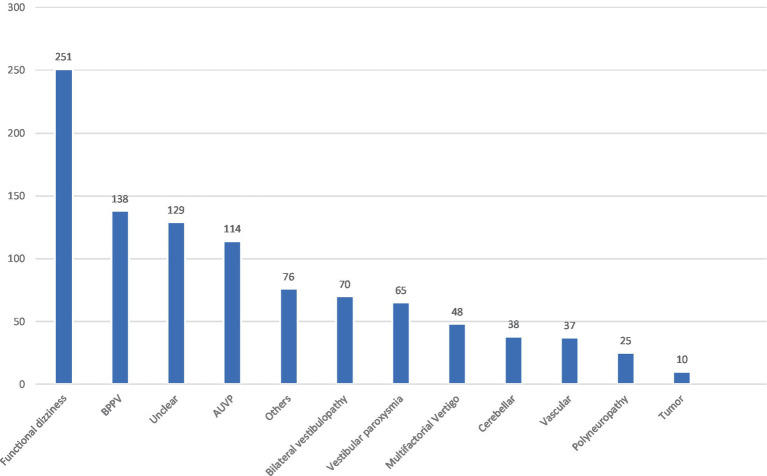
Different vertigo entities in the comparison group. BPPV, benign paroxysmal positional vertigo; AUVP, acute unilateral vestibulopathy (including residual vertigo/dizziness in the post-acute phase).

In this retrospective study, a total of 2,101 patients were included between January 2010 and February 2020: 2,020 subjects from the Ludwig Maximilian University (LMU) Hospital Munich, Germany; 25 from the Sint-Jan Clinic in Bruges, Belgium; and 56 from the Maastricht University Medical Center, Netherlands. The study was approved by the institutional ethics review boards (No. 19-301) The study group consisted of 1,100 patients who fulfilled the diagnostic criteria defined by the Bárány Society for certain (*n* = 374) or probable (*n* = 253) MD or certain (*n* = 142) or probable (*n* = 331) VM (1, 2). The comparison group consisted of 1,001 patients with various central, peripheral, and functional vertigo disorders (Table 1; Figure 1). All subjects had a complete diagnostic work-up, including caloric and vHIT testing.

**Table 1:** Demographic and clinical characteristics.

Characteristics	*N* (%)/Median (range)		
	Menière’s disease	Vestibular migraine	Other vestibular disorders
*N*	627	473	1,001
Sex			
Men	325 (51.8%)	166 (35.1%)	444 (44.3%)
Women	302 (48.2%)	307 (64.9%)	557 (55.6%)
Age	58.2 ± 14.5 (11–88)	46.8 ± 14.4 (5–84)	55.0 ± 16.6 (9–95)
Certainty of diagnosis^1^			
“Diagnosis of …”	374 (59.6%)	142 (30.0%)	
“Probable diagnosis of …”	253 (40.3%)	331 (70.0%)	
Right	253 (40.3%)		
Left	260 (41.5%)		
Bilateral MD	114 (18.2%)		
Pathological vHIT (gain on either side <0.7)	101 (16.1%)	25 (5.3%)	240 (24%)
Pathological caloric testing^2^	446 (71%)	99 (20.9%)	333 (33.3%)
Normal vHIT and pathological caloric testing	369 (58.9%)	78 (16.5%)	165 (16.5%)

1 Barany Society criteria (2015), Vestibular migraine. Diagnostic criteria (2012).

2 A variability of ≥25% and/or a total caloric excitability of <10°/s in both ears was considered pathological.

vHIT, video head impulse test.

### 2.1 Video head impulse testing

The vHIT was performed using the device “Otometrics^®^” with a visual target fixation distance of 1.8 m and a peak velocity horizontal plane >150°/s. The device consists of a headset that uses an accelerometer and a camera mounted on a set of goggles to measure head and eye movement. The patients were instructed to stare at a target positioned at eye level, and several passive quick head rotations were performed by the examiners. Ideally, the head movements are accompanied by eye movements that are equal in velocity and opposite in direction. This is then described as an eye/head gain of 1.0 (25). An impaired vestibulo-ocular reflex (VOR) causes a reduced acceleration of the eyes, resulting in a lower gain than 1 with catch-up overt or covert saccades (26). A vHIT gain ≥0.7 was considered normal (27).

### 2.2 Caloric testing

Caloric testing was performed using “Atmos Variotherm^®^” as a caloric water stimulator and “Interacoustics VOG^®^” for recording eye movements. The caloric testing relies on the application of cold and warm water in the external ear canal. The differences in temperature cause the endolymphatic liquid in the horizontal semicircular canal to move. This results in a calorically induced nystagmus, whose slow phase is then measured by a camera, mounted on a set of goggles. The VOR frequencies evaluated by the caloric stimulation are within the range of 0.003–0.008 Hz, which is lower than those of the vHIT (28). The irrigations were performed with a minimum of 100 mL of water for a duration of 30 s. The interval between the first irrigation and the following irrigation was 300 s. The cold stimulation was performed at 30°C, and the warm stimulation was performed at 44°C. Unilateral weakness or canal paresis was calculated according to Jongkees’ formula; a variability of ≥25% was considered pathological. A bilateral canal paresis was defined as a reduced total slow phase velocity of the warm and cold stimuli of less than 10°/s (27).

### 2.3 Data analysis

Statistical analysis was performed using SPSS. Categorial data were expressed as numbers (%), and continuous values were expressed as median and range. To assess whether the discrepancy between a normal vHIT and a reduced caloric excitation can serve as a marker for MD, we calculated the sensitivity, specificity, positive predictive value (PPV), and negative predictive value (NPV) and compared the individual groups with each other (MD vs. VM, MD vs. comparison group, and MD vs. comparison group + VM). Furthermore, we assessed the diagnostic significance of caloric testing only. Receiver operating characteristic (ROC) curves were used to compare the diagnostic value of a normal vHIT and a reduced caloric excitation with pathological caloric testing alone. Comparisons of sensitivity/specificity between the two methods (normal vHIT + reduced caloric excitation vs. reduced caloric excitation only) were performed using the McNemar test for paired samples. A *p*-value of <0.05 was considered statistically significant.

## 3 Results

A total of 627 patients with MD and 473 patients with VM were included (Table 1). In MD, 59.6% of the patients met the diagnostic criteria for “definite MD” and 40.3% for “probable MD.” The median age in the MD group was 58 years, and the gender distribution was almost equal, with 51.8% men and 48.2% women. In the VM group, only 30% of the patients were classified as “VM” and 70% as “probable VM.” The median age was 46.8 years, and the majority were women (64.9%). The comparison group consisted of 1,001 patients with various other vestibular disorders; at least one episode of vertigo or persisting dizziness was required for inclusion. The details of the comparison group are given in Figure 1.

The McNemar test was used to determine the statistical significance of the result in the 2 × 2 contingency tables depending on the analysis of paired data.

### 3.1 Diagnostic value of normal vHIT and pathological caloric testing (dissociation) for identifying patients with MD among other vestibular disorders

In the MD group, 369 patients (58.9%) showed a discrepancy between the vHIT and caloric testing, with normal vHIT (gain >0.7), while caloric testing yielded asymmetric results (>25% and/or total caloric excitation <10°/s for one side). Thus, the sensitivity for identifying MD patients via a discrepant vHIT and caloric testing, what we will call “dissociation” in the following, was 58.9% (Table 2). Compared to the comparison group, the proportion of false-positive findings was 16.5%, defining the specificity at 83.5%. The PPV was 69.1%, and the NPV was calculated at 76.4% (*p* < 0.001, McNemar test).

**Table 2:** Sensitivity and specificity of normal vHIT and pathological caloric testing for identifying MD among other vertigo entities.

	Sensitivity (%)	Specificity (%)	PPV (%)	NPV (%)	*p*-value
MD vs. CP	58.9	83.5	69.1	76.4	<0.001^a^
MD vs. VM	58.9	83.5	82.6	60.5	<0.001^a^
MD vs. VM + CP	58.9	83.5	60.3	82.7	<0.001^a^

MD, Menière’s disease; CP, comparison group; VM, vestibular migraine; ^a^McNemar Test.

### 3.2 Diagnostic value of dissociation for identifying patients with MD vs. patients with VM

**Figure 2:**
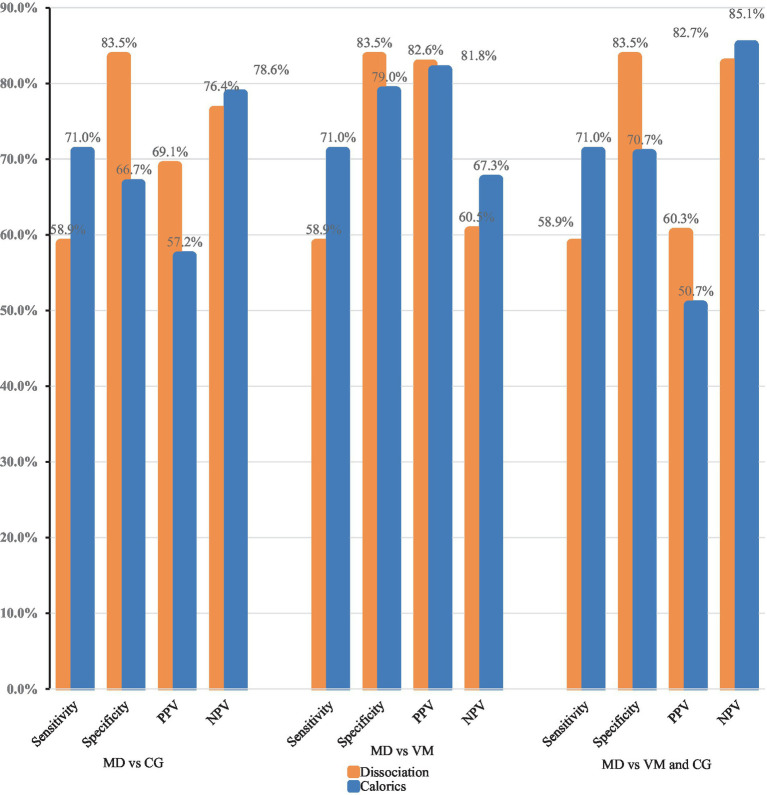
Comparison of the diagnostic power of a normal video head impulse test and a pathological caloric excitation (dissociation) to caloric testing alone. PPV, positive predictive value; NPV, negative predictive value; MD, Menière’s disease; CG, comparison group; VM, vestibular migraine.

In the VM group, the specificity of the discrepancy was 83.5% (395 out of 473 patients). Due to the low false-positive rate, the PPV was 82.6% (Table 2) and the NPV was 60.5% (Figure 2).

### 3.3 Diagnostic value of dissociation for identifying MD patients vs. all other vestibular disorders (comparison group plus VM)

The following results were obtained by comparing the diagnostic value of a normal vHIT and pathological caloric testing to identify MD patients among other vestibular disorders (comparison group plus VM): The sensitivity and specificity remained the same at 58.9 and 83.5%, respectively. The NPV was 82.7%, and the PPV was 60.3%.

### 3.4 Diagnostic value of caloric testing alone for identifying MD patients

Among MD patients (*N* = 627), caloric testing was pathological in 71%. Overall, 20.9% of the patients in the VM group and 33.3% of the patients in the comparison group showed a reduced caloric response. The specificity of caloric testing alone was significantly lower when compared to the specificity of a normal vHIT and pathological caloric testing (Tables 2, 3). When comparing MD and VM, the sensitivity of caloric testing was 71% and the specificity was 79% (PPV: 81.8% and NPV: 67.3%). The specificity for identifying MD patients among other vertigo entities (comparison group) via caloric testing was lower at 70.7%.

**Table 3:** Sensitivity and specificity of caloric testing only for identifying MD among other vertigo entities.

	Sensitivity (%)	Specificity (%)	PPV (%)	NPV (%)	*p*-value
MD vs. CG	71	66.7	57.2	78.6	<0.001^a^
MD vs. VM	71	79	81.8	67.3	<0.001^a^
MD vs. VM + CP	71	70.7	50.7	85.1	<0.001^a^

MD, Menière’s disease; CG, comparison group; VM, vestibular migraine; ^a^McNemar test.

### 3.5 Caloric testing vs. caloric testing and vHIT for identifying MD patients

**Figure 3:**
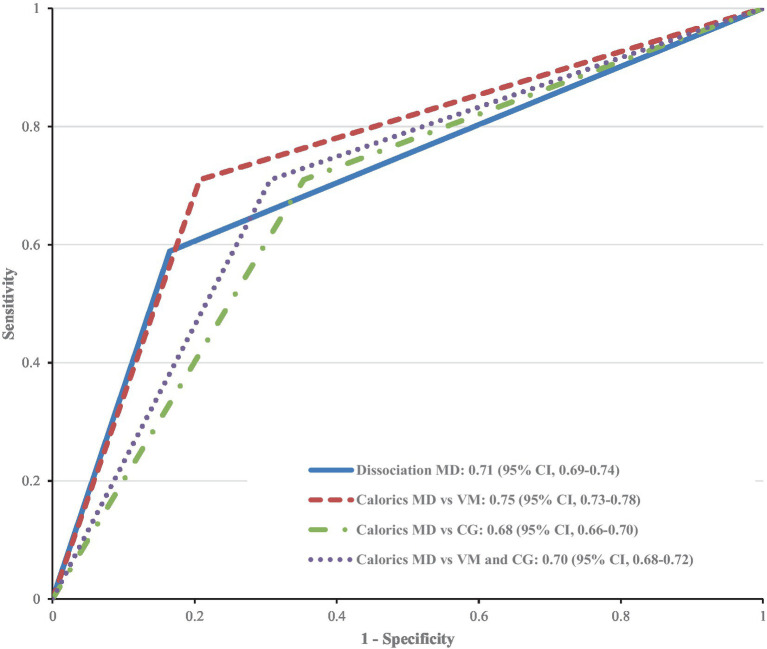
Receiver operating characteristic (ROC) curve for the diagnostic power of pathological caloric testing alone vs. pathological caloric testing combined with a normal video head impulse test (dissociation) in the diagnosis of: MD vs. VM, MD vs. other vestibular disorders (comparison group CG), and MD vs. VM and comparison group. The ROC curve and the area under the curve are the same when it comes to using the dissociation as a test to diagnose MD vs. VM; MD vs. other vestibular disorders, and MD vs. VM and others.

It was also assessed whether a dissociation of vHIT and caloric testing (normal vHIT vs. pathological caloric testing) has a higher diagnostic value than caloric testing alone. Due to their low sensitivity (58.9% vs. 71%), both tests are quite unsuited for screening patients without typical clinical symptoms. We illustrated the receiver operating characteristics curve (ROC curve, Figure 3) to depict the diagnostic power of the two tests. The caloric testing showed the largest area under the curve when diagnosing MD vs. VM (0.75, 95% CI, 0.73–0.78). This is in line with the fact that the dissociation consists of two paired diagnostic tests, thus delivering a lower sensitivity. However, the combination of caloric testing and vHIT ensures a higher specificity when a dissociation is present (83.5% vs. 66.7%, *p* < 0.001). Accordingly, a dissociation of vHIT and caloric testing can serve as a rule-out test and has a higher diagnostic value than caloric testing alone.

## 4 Discussion

In the confirmatory part of this retrospective analysis in a large patient cohort (*N* = 2,101), the diagnostic value of the—well-known—discrepancy between a pathological caloric excitation and a normal vHIT test in patients with MD was analyzed. Considering the differentiation between MD and VM, the discrepancy was highly specific for MD (83.5%). Together with a low percentage of false-positive results and a high positive predictive value (82.6%), it can be used as an assisting rule-out test for MD—especially in patients lacking the typical MD symptoms in the early stages of the disease.

When the working diagnosis included distinguishing between MD and other vestibular disorders, the discrepancy remained highly specific for MD (83.5%). A higher PPV (69.1%) and a similar NPV (76.4%) made the dissociation the better MD exclusion test.

Recently, with the dissociation getting more attention from researchers, several theories have been introduced as to what the pathophysiological mechanism behind the dissociation might be. MD might affect regular and irregular afferents differently, leading to a loss of type II hair cells in the crista ampullaris starting peripherally (23, 29). The peripheral zones might be more sensitive to low-frequency regular afferent excitation, which is performed in a caloric test, leading to pathological results. The high-frequent irregular afferents, located centrally, are thought to be damaged by the disease in much later stages—they are tested by the vHIT test, which often leads to a normal result of the test. Some reasonable doubt regarding the theory can be expressed due to the observation of type I and II hair-cell loss as well as basal membrane damage in patients with MD (30). A second, more widely distributed theory is the one explaining the pathophysiology of MD with a physical hydropic enlargement of the membranous duct, also known as endolymphatic hydrops (22).

In conclusion, the discrepancy between a normal vHIT test and pathological caloric excitability is a useful parameter showing high specificity for patients suffering from MD. It offers better diagnostic power than vHIT and caloric testing taken separately and requires a little more effort to investigate, especially for patients who receive both tests as a first-line diagnostic tool. The dissociation proves to be a relevant MD exclusion test in the differential diagnostics of MD against various vestibular disorders, not only vs. VM, on which the research has been focused so far. The quantification of this discrepancy and whether it reflects the current MD stage or moments of evaluation—non-ictal vs. ictal—may be of interest to future research. Setting optimal values for pathological caloric and video head impulse testing should also be considered, namely due to the larger variability from center to center (27).

## 5 Limitations

As a retrospective study, our study has certain limitations. Despite our efforts to record a large number of subjects’ data as objectively as possible, misclassification and selection biases can never be fully excluded and can lead to skewed results and untrue or incomplete conclusions. A further limitation that needs to be pointed out is the lack of a control group consisting of patients without dizziness to control the study, which could help further investigate the diagnostic power of the described dissociation. The primary goal of the study was to provide more insight and assistance to diagnosis-making in an everyday clinical setting where usually the patients with some form of dizziness are going to be the ones who receive a video-head-impulse test and caloric testing. We aimed to help clinicians more efficiently interpret the two tests in an environment where the tests have already been carried out, as opposed to using the dissociation as a form of screening test and determining its presence in healthy individuals. Considering this, we did not include subjects without vertigo or dizziness as a part of this study. To diminish the aforementioned limitations, a further prospective blinded study design with the inclusion of a control group of healthy individuals is required.

## Data Availability

The raw data supporting the conclusions of this article will be made available by the authors, without undue reservation.
